# Niagara and Orleans County Veterinary Medical Society

**Published:** 1896-12

**Authors:** J. P. Thomson

**Affiliations:** Secretary


Secretary.

### NIAGARA AND ORLEANS COUNTY VETERINARY MEDICAL SOCIETY.

A MEETING was held at Medina, New York, August 18, 1896, Dr. Martin in the chair. Members present: Drs. Martin, Stocking, Thomson, Hunter, Kesler, Williams, Moore, and Crowforth.

Minutes of last meeting read and approved. Report of the By-laws Committee was received and ordered laid on the table until later in the meeting. Moved and seconded that the organization be known under the name of the Niagara and Orleans County Veterinary Medical Society; carried. Moved and seconded that the Secretary procure a copy of all laws pertaining to veterinary practice enacted since 1886, and report at next meeting; carried. Moved and seconded that the Secretary, Dr. J. P. Thomson, act in concert with the Committee on By-laws, to confer with Dr. Hinkley, and draft by-laws to submit at the next meeting; carried. Moved and seconded that the amount of initiation fees and dues be left blank until the Secretary investigate into the cost of printing by-laws and certificates similar to that of the New York State Veterinary Medical Society. Moved and seconded that we adjourn to meet at the call of the Secretary between the 1st and 10th of October, at the Niagara House, Lockport, New York, at 1.30 P M. ; carried. Meeting adjourned. J. P. THOMSON,
Secretary.

At the meeting of the Niagara and Orleans County Veterinary Medical Society on the 9th of October, the further consideration of the adoption of a constitution and by-laws were considered, after which the meeting adjourned to convene at Niagara Falls in January, 1897.

